# Addressing abdominal trauma from conflict and warfare in under‐resourced regions: A critical narrative review

**DOI:** 10.1002/hsr2.70151

**Published:** 2024-10-23

**Authors:** Wireko Andrew Awuah, Joecelyn Kirani Tan, Muhammad Hamza Shah, Arjun Ahluwalia, Sakshi Roy, Syed Hasham Ali, Tomas Ferreira, Hareesha Rishab Bharadwaj, Favour Tope Adebusoye, Nicholas Aderinto, Adele Mazzoleni, Toufik Abdul‐Rahman, Denys Ovechkin

**Affiliations:** ^1^ Faculty of Medicine Sumy State University Sumy Ukraine; ^2^ Faculty of Medicine University of St Andrews St. Andrews Scotland UK; ^3^ Faculty of Biology, Medicine and Health The University of Manchester Manchester UK; ^4^ School of Medicine Queen's University Belfast Belfast UK; ^5^ Faculty of Medicine, Dow Medical College Dow University of Health Sciences Karachi Pakistan; ^6^ Bristol Medical School University of Bristol Bristol UK; ^7^ Department of Internal Medicine LAUTECH Teaching Hospital Oyo Nigeria; ^8^ Faculty of Medicine, Barts and the London School of Medicine and Dentistry UK

**Keywords:** abdominal surgery, abdominal trauma, conflict, emergency care, Global surgery, low and middle‐income countries

## Abstract

**Introduction:**

The prevalence of abdominal injuries in war and conflict zones, particularly in low‐ and middle‐income countries (LMICs), presents a significant healthcare challenge. These injuries, often resulting from explosive devices, firearms, and shrapnel, lead to high morbidity and mortality rates due to delayed diagnoses, inadequate medical infrastructure, and limited access to specialised care. This review aims to summarise the literature on conflict‐related abdominal injuries in LMICs, highlighting the impact of such trauma on healthcare systems and patient outcomes, and suggesting strategies for improvement.

**Methods:**

A comprehensive narrative review was conducted, focusing on studies from contemporary and historical conflict‐ridden nations. Databases such as PubMed, EMBASE, Google Scholar, the Cochrane Library, and Scopus were searched using specific keywords. Inclusion criteria encompassed various study designs and both paediatric and adult populations, with studies providing raw data prioritised. Exclusions included non‐English articles, non‐peer‐reviewed studies, and those not reporting outcomes or involving high‐income countries.

**Results:**

The review identified significant challenges in managing war‐related abdominal trauma in LMICs, including a shortage of healthcare personnel and infrastructure, socio‐political barriers, and research gaps. Clinical implications of such injuries include elevated mortality rates, with surgical and nonsurgical management outcomes varying significantly. Positive advancements in diagnostics and surgical techniques have improved survival rates, yet the need for further infrastructural and workforce enhancements remains critical.

**Conclusion:**

Abdominal trauma in conflict‐affected LMICs necessitates focused efforts to improve healthcare delivery, including targeted funding for infrastructure and equipment, development of training programs for trauma specialists, and increased humanitarian aid. Bridging research gaps and fostering collaborative efforts are essential for advancing the management of abdominal trauma and enhancing patient outcomes in these challenging environments.

## 1 INTRODUCTION

An estimated 4400 fatalities occur daily due to acts of violence.^1^ This statistic, however, fails to account for nonlethal injuries and their impacts on patients’ families. It is also important to note that previous literature might have underestimated the mortality caused by war and conflict, as recent data suggest a significant number of indirect victims in the aftermath of such conflicts.^2^

Among the variety of injuries associated with war and conflict, those inflicted on the abdominal region are particularly severe. Such trauma, induced by mechanisms including but not limited to gunshot wounds, blast injuries, and explosive device fragmentations, can either penetrate or cause blunt damage.^3^, ^4^ These injuries’ characteristics and severity depend on the nature of the conflict, the weaponry involved, and the injury context, varying from superficial to complex multiorgan and structural injuries.^5^ Notable features of conflict‐induced abdominal trauma include extensive tissue damage, internal bleeding, visceral injuries, viscus perforations, solid organ damage, and vascular complications.^4^ The timely provision of specialised medical attention is often critical for these patients, as failure to intervene promptly can result in life‐threatening complications such as death, sepsis, and severe haemorrhage. However, in most low‐ and middle‐income countries (LMICs), healthcare deficits are exacerbated by war and conflict, resulting in escalated complications and mortality.^6^, ^7^

The predominant use of explosive devices, firearms, and shrapnel, along with limited access to healthcare services, inadequate medical infrastructure, and delayed medical intervention, primarily contribute to the increased incidence of war‐induced abdominal trauma.^6^ Clinical presentations of abdominal trauma in war zones exhibit distinctive characteristics compared to nonwar settings. The urgency and resource‐constrained environments prevalent in conflict zones pose unique challenges for medical professionals in assessing and managing abdominal injuries. Limited access to advanced diagnostic tools like computed tomography (CT) scanners or interventional radiology, coupled with a scarcity of skilled healthcare personnel, compound these challenges.^6^ Consequently, such trauma may result in delayed or missed diagnoses, increased complication rates, and heightened mortality in comparison to nonwar settings.^6^

The determinants of outcomes in abdominal trauma cases are multifaceted, involving patient, surgeon, anaesthetic, and injury factors. Patient factors such as age and pre‐existing health conditions significantly impact recovery and survival rates.^8^ Surgeon expertise and the availability of specialised training in trauma care also play crucial roles in determining outcomes.^9^ The choice and administration of anaesthetics can affect intraoperative stability and postoperative recovery, with deviation from surgical and anaesthetic care plans being associated with increased risk of adverse events.^10^ The nature and severity of the injury itself, including the mechanism of trauma and the extent of organ damage, are critical determinants of both morbidity and mortality.^11^

Particularly in LMIC conflict settings, war‐induced abdominal trauma significantly strains healthcare systems. In war zones, studies indicate that abdominal trauma accounts for ~18% to 25% of injuries, compared to non‐conflict regions where abdominal trauma comprises roughly 5% of total injuries.^7^ Understanding this disparity is critical for resource allocation, trauma preparedness, and targeted interventions in conflict zones.^12^ The general pattern of abdominal injuries in conflicts and wars in LMICs reveals a predominance of penetrating injuries due to gunshots, shrapnel, and explosions.^6^ These injuries often result in complex, multiorgan damage requiring extensive surgical intervention and prolonged recovery periods.^5^ Non‐penetrating injuries, although less common, also occur due to blunt force from blasts or structural collapses.^3^, ^4^ Recognising the specific needs of conflict‐affected populations, healthcare providers and policymakers can develop strategies to mitigate the impact of conflict on abdominal injuries and improve patient outcomes.^12^ By addressing both the immediate surgical needs and the long‐term rehabilitation requirements, healthcare systems can better manage the surge in abdominal trauma cases during conflicts.

To the best of our knowledge, this review is the first to summarise the literature on conflict‐related abdominal injuries in LMICs. Its principal objective is to comprehensively analyse the existing literature on the subject to better understand the definition, aetiology, clinical manifestations, and prevalence of this specific injury type in both contemporary and historical conflict‐affected nations. This review aims to identify current knowledge gaps, suggest potential interventions, and ultimately facilitate the development of effective prevention, management, and rehabilitation strategies in conflict‐affected populations.

## 2 METHODOLOGY

This narrative review adopts a comprehensive approach to assessing the management of abdominal trauma in conflict‐affected regions. A systematic search of published literature was conducted, focusing particularly on studies undertaken in contemporary and historical conflict‐ridden nations and those populations directly impacted, whether within or outside the immediate conflict zone.

The scope of this review encapsulates diverse forms of conflict, including but not limited to civil wars, communal unrest, organised terroristic and rebellious activities, inter‐nation wars (either via proxy or direct engagement), territorial disputes, and separatist conflicts. Excluded are incidents precipitated by generalised lawlessness or judicial laxity, such as armed robberies, muggings, and interpersonal altercations.

‘Abdominal injury’ or ‘trauma’ within this review is defined as any physical harm to structures within the abdominal cavity or the overlying dermis resulting from external force arising from violent encounters. This includes but is not limited to damage to organs, blood vessels, muscles, and other tissues within the abdominal region due to varied mechanisms like blunt or penetrating trauma, explosive devices, firearms, or other forms of violence. Such injuries can range from minor contusions and superficial lacerations to severe fractures, perforations, organ ruptures, vascular damage, or internal bleeding. Treatment and diagnostic outcomes as reported by each study are documented.

The inclusion criteria encompass various study designs, including descriptive, case‐control, cohort, observational, and randomised controlled trials. Both current and historical studies of conflict‐ridden nations are considered eligible, as are studies involving comorbidity and prior pharmacological or surgical treatments. Both paediatric and adult populations are considered, and only full‐text articles in English are included. Studies not including reported outcomes or with populations native to high‐income countries are excluded.

Databases such as PubMed, EMBASE, Google Scholar, the Cochrane Library, and Scopus were employed in this review. Search terms included keywords such as “abdominal trauma”, “abdominal injury”, and “management”, combined with indicators such as “conflict‐affected”, “war‐torn”, “conflict” and “war”. Additional sources were identified via a manual search of references from recent disease‐specific reviews. Excluded from this review were stand‐alone abstracts, case reports, posters, and unpublished or non‐peer‐reviewed studies.

No strict sample size requirement was imposed to ensure the inclusion of literature from smaller centres with fewer admissions for abdominal trauma and injury cases. Only studies providing raw data were included; those with estimated or modelled numerator or denominator values were excluded.

The review aimed to provide a comprehensive academic evaluation of the management of abdominal trauma and injury through a robust approach to synthesising data. A summary of the methodology employed is presented in Table 1.

**Table 1:** Summary of Methodology for this Review.

Methodology steps	Description
Literature search	Utilised databases: PubMed, EMBASE, Google Scholar, the Cochrane Library, and Scopus.
Inclusion criteria	Studies published in English. Descriptive, case‐control, cohort, observational, and randomised controlled trials. Studies from both contemporary and historical conflict‐ridden nations. Involving comorbidity and prior treatments. Covering both paediatric and adult populations. Full‐text articles only. Studies providing raw data.
Exclusion criteria	Incidents of generalised lawlessness or judicial laxity (e.g., armed robberies, muggings). Stand‐alone abstracts, case reports, posters. Unpublished or non‐peer‐reviewed studies. Studies not including reported outcomes or native to high‐income countries. Studies with estimated or modelled data.
Search terms	Keywords such as “abdominal trauma”, “abdominal injury”, and “management” combined with “conflict‐affected”, “war‐torn”, “conflict”, and “war”.
Additional search	Manual search of references from recent disease‐specific reviews, without a strict sample size requirement to include literature from smaller centres.

## 3 EVALUATING CHALLENGES WITH WAR‐ RELATED ABDOMINAL INJURY IN LMICS

### 3.1 Limited availability of emergency and trauma care staff

The presence of an adequate number of healthcare professionals in conflict zones is crucial. It directly influences the ability to deliver essential medical services, alleviate suffering, and optimise outcomes for abdominal trauma victims. Experiences from conflict zones over recent decades have highlighted the formidable challenge of maintaining an adequate supply of healthcare personnel in these regions due to several hindrances.

Firstly, the risk to personal safety has deterred numerous healthcare professionals from practising in these areas. The group ‘Physicians for Human Rights’, for instance, reports that over 800 healthcare workers, many of whom provide trauma and surgical care, have been killed in the Syrian conflict since 2017.^13^ Similar statistics have emerged from global conflicts in Iraq, Libya, Somalia, Pakistan, and South Sudan.^14^

Secondly, exacerbating the problem is the mass migration of health workers from conflict zones. Civil and regional wars in countries such as Lebanon, Somalia, Liberia, Iran, and Afghanistan have led to the mass exodus of trauma care personnel, further impairing the delivery of care for abdominal trauma victims.^15^ In Syria, for instance, over 70% of healthcare workers sought refuge and asylum elsewhere.^16^ This migration is influenced not only by the conflicts themselves but also by poor working conditions and inappropriate financial compensation.^17^

Thirdly, healthcare professionals in conflict zones often encounter patients with delayed presentations, which typically increase the complexity of the cases.^18^ Combined with staff shortages, the absence of formal supervision and training, and recurrent disruptions to basic services such as water and electricity, this can compromise workforce capability and job satisfaction.^19^, ^20^, ^21^, ^22^, ^23^, ^24^ Additionally, despite evidence supporting the critical need for abdominal trauma care staff in conflict zones, these compounding factors often result in severe shortages.

### 3.2 Acute shortage of general and specialised healthcare infrastructure for effective abdominal trauma management

The dearth of resources within medical institutions in conflict zones responsible for abdominal trauma care is concerning. Institutions with long‐term experience in managing such trauma in conflict‐affected regions consistently highlight the urgent need for prompt diagnosis, particularly for penetrating abdominal injuries.^25^

Multiple studies, centred on abdominal trauma cases within conflict regions, affirm the persistent shortage of diagnostic facilities. For instance, hospitals in the conflict‐affected regions of Nigeria reportedly lack essential imaging instruments such as CT and MRI scanners, indispensable for precise abdominal trauma diagnosis.^18^ Further investigations conducted in various conflict‐affected regions of Western Africa describe how insufficient funding has resulted in defunct or underperforming equipment, significantly impacting the reliability and accuracy of diagnoses.^18^, ^26^, ^27^ An observable disparity exists in the distribution of diagnostic resources, with civilian hospitals enduring shortages while military bases, due to superior funding, have improved access to CT and FAST scanners, thereby enabling improved diagnosis of abdominal traumas.^18^, ^28^ This generalised shortage of diagnostic equipment is further exacerbated by the limited availability of surgical equipment, hindering the surgical staff's ability to conduct serial clinical and radiological assessments of affected organs and thereby obstructing surgical treatment provision.^29^

Challenges arising from inadequate diagnosis lead to further difficulties in delivering effective treatment, resulting in worsened patient outcomes. For instance, Cardi et al. reported that the lack of diagnostic equipment and surgical supplies in areas affected by the Taliban insurgency in Afghanistan hindered patient triage, caused diagnostic delays, and complicated the management of abdominal trauma cases.^30^

Reports from conflict‐affected regions, including specific areas of Afghanistan, highlight inadequate access to critical care facilities, blood products, and specialised wound care materials within local hospitals.^31^, ^32^, ^33^ This scarcity of essential resources further complicates the management of abdominal trauma cases, thereby amplifying the challenges confronted by healthcare providers.^31^ Significant disparities in the availability of these resources exist, with contrasting situations in different conflicts. For instance, during the Afghanistan conflict, there were reported shortages of blood products for civilians, in stark contrast to the excess supply for US Army victims during the Vietnam War.^32^ Despite several studies consistently evidencing the mortality reduction benefit associated with prompt diagnosis and the availability of advanced life support materials for war‐related abdominal trauma, many regions continue to lack access to such facilities.^33^

### 3.3 Socio‐Political barriers to care

International humanitarian law, dating back approximately 150 years, unambiguously dictates that entities engaged in any form of conflict—be it regional, civil, or international—must not target healthcare workers and facilities. However, a distressing trend has emerged in conflict zones, with recurring transgressions committed against healthcare personnel and institutions in violation of these norms.^14^ Over 430 instances of hospital‐targeted violence have been documented by the Global Terrorism Database, leading to an excess of 1291 lives lost.^34^ Such assaults are compounded by the destruction of medical equipment, seizure of hospitals by military forces, and pillaging of medical facilities, significantly impairing the delivery of care for abdominal trauma patients.^14^, ^35^

Various factors, such as the presence of landmines and continued armed conflict, deter patients from seeking treatment for abdominal traumas, as demonstrated in Afghanistan.^30^ Additionally, reports suggest deliberate obstructions to healthcare access, with certain factions intentionally impeding or delaying patients’ access to hospitals and healthcare institutions. For instance, insurgent groups in South Sudan have been known to impede local residents from obtaining healthcare in Darfur, with similar instances reported in Iraq, Syria, and the Central African Republic.^14^

The diversion of significant amounts of funding away from healthcare towards militaristic endeavours due to ongoing wars and conflicts presents another significant socio‐political barrier to healthcare accessibility. Several studies indicate that this financial redirection precipitates a critical shortage of essential surgical equipment and supplies necessary for trauma care, exacerbating the challenges faced by healthcare workers.^15^, ^35^

Reports also indicate instances where groups have hindered the delivery of medical aid from Nongovernmental organisations (NGOs) and charities, as seen in South Sudan and certain regions of Turkey.^14^ Collectively, these socio‐political factors pose significant obstacles to the provision of surgical care for abdominal traumas and highlight an urgent area of concern.

### 3.4 The presence of significant research gaps

In addition to the above, substantial research gaps pose additional challenges in evaluating the scale of abdominal trauma in conflict‐affected regions and assessing the nature of care available to its victims. A major barrier lies in acquiring circumstantial data. As evidenced by studies exploring the Syrian conflict, understanding the cause of the abdominal trauma remains crucial to guide management decisions, yet it remains challenging to obtain from patients.^6^ Additionally, limited access to conflict zones, security concerns, and logistical constraints pose significant challenges in conducting research and collecting data on abdominal traumas in war‐torn areas.^15^, ^35^

The lack of standardised data collection protocols and the variability in healthcare systems across different conflict settings undermine the comparability and generalisability of studies, consequently limiting the comprehensive understanding of the specific challenges and conditions associated with abdominal trauma.^36^, ^37^, ^38^

Inadequate funding and a low prioritisation of research in conflict‐affected regions further exacerbate these research gaps, obstructing efforts to comprehensively address the management and outcomes of abdominal trauma. Despite numerous conflicts erupting since the turn of the millennium, studies reporting the outcomes of abdominal trauma are largely confined to a handful of regions. The dearth of research on conflict‐related abdominal trauma in certain conflict regions, including Kyrgyzstan, Libya, Sudan, and others, obscures our understanding of the specific challenges and circumstances associated with abdominal trauma within these settings, skewing the overall narrative. The challenges in abdominal trauma management in conflict‐affected LMICs have been summarised in Table 2.

**Table 2:** Challenges in Abdominal Trauma Management in Conflict‐Affected Low and Middle Income Countries.

Challenge category	Specific challenges	Explanation
Staff availability and safety concerns^13^, ^14^, ^15^, ^16^, ^17^, ^18^, ^19^, ^20^, ^21^, ^22^, ^23^, ^24^	Risk to personal safety. Healthcare worker migration. Delayed patient presentations. Shortages and lack of training. Service disruptions.	The combination of personal safety risks, mass migration, and service disruptions leads to severe shortages of healthcare workers, affecting the delivery of care for abdominal trauma victims in conflict zones.
Healthcare infrastructure deficits^18^, ^25^, ^26^, ^27^, ^28^, ^29^	Diagnostic and surgical equipment shortages. Funding inadequacies. Resource distribution disparities. Diagnostic and treatment challenges.	Significant shortages in essential medical infrastructure, particularly diagnostic and surgical tools, are exacerbated by funding issues and resource distribution disparities, impairing abdominal trauma management.
Socio‐political impediments^14^, ^30^, ^31^, ^32^, ^33^, ^34^, ^35^	Targeting of healthcare facilities. Landmines and ongoing conflict. Diversion of healthcare funding. Access obstructions to healthcare.	Socio‐political barriers, including violence against healthcare settings, funding redirection to military efforts, and deliberate healthcare access obstructions, significantly hinder abdominal trauma care provision.
Research and data collection challenges^6^, ^15^, ^35^, ^36^, ^37^, ^38^	Data acquisition barriers. Conflict zone access issues. Non‐standardised data protocols. Funding and prioritisation shortfalls.	The difficulty in obtaining accurate data, combined with logistical and security challenges in conflict zones, undermines research efforts and exacerbates the knowledge gap in managing abdominal trauma effectively.

## 4 CLINICAL IMPLICATIONS OF ABDOMINAL TRAUMA IN WAR AND CONFLICT LMICS

### 4.1 Impact of abdominal trauma in war and Conflict‐Affected LMICs on the general population: Mortality, quality of life, and healthcare needs

Abdominal trauma management in conflict‐affected nations presents significant challenges, often leading to severe health outcomes, including elevated morbidity and mortality rates. Research highlights the critical nature of abdominal injuries in battlefield settings, where factors like irreversible shock, extensive organ damage, severe head injuries, and delayed medical care significantly increase mortality risks. Despite advanced resuscitation efforts, mortality rates remain high, underscoring the urgent need for immediate medical access and coordinated trauma care systems.^4^ The nature of the trauma, such as accidental, self‐inflicted, or assault‐related, markedly affects outcomes, with self‐inflicted injuries resulting in notably worse health‐related quality of life compared to other types.^39^ While trauma systems have shown potential in improving survival rates, their impact on morbidity, quality of life, and economic burdens requires further exploration.^40^

A study from Baghdad during the COVID‐19 pandemic identified small bowel and shrapnel injuries as common in penetrating abdominal trauma, with gunshot wounds, organ damage extent, delayed hospital arrival, and blood transfusion volume as key mortality factors.^41^ Similarly, research focused on Yemen, highlighted the prevalence of blunt abdominal trauma (BAT), which accounts for 7–10% of trauma cases, emphasising the challenges in diagnosing and managing injuries to both intraperitoneal and retroperitoneal organs.^42^ Despite advancements in imaging and medical technology, in‐hospital mortality rates for trauma, especially BAT, have not significantly improved, with delayed care, injury severity, and significant brain injuries as major determinants of poor outcomes.^43^ The rise in abdominal injuries from civilian conflicts, driven by armed robbery, communal clashes, political unrest, and cultism, is alarming, with factors like advanced age, gunshot wounds, delayed care, bowel resection necessity, and multiple injuries escalating mortality rates.^42^

### 4.2 Outcomes of abdominal trauma management in conflict and war zones ‐ surgical and nonsurgical interventions and mortality rates

Surgical management of abdominal trauma can lead to significant adverse effects, including wound infections, intra‐abdominal abscesses, faecal fistulas, hypovolemic shock, cardiac injuries, aortic injuries, and sepsis.^36^, ^37^, ^44^ Complications and morbidities such as septicemia, HIV infection, haemorrhagic shock, and visceral organ injuries are linked to increased mortality rates.^36^, ^37^, ^44^ Research from war‐ and conflict‐affected regions reports a consistent mortality rate of at least 20% for abdominal trauma management, underscoring the need for specialised protocols and guidelines in these resource‐limited settings.^45^, ^46^, ^47^, ^48^, ^49^, ^50^

In Iraq, the prevalence of abdominal traumas, mainly from explosive devices, presents significant management challenges due to medical infrastructure limitations and a high risk of postoperative infections.^51^ Similarly, the Syrian conflict has introduced unique management obstacles for abdominal injuries, including high complication rates from delayed interventions and medical resource scarcity, significantly influencing mortality rates.^52^ A study emphasised the importance of medical staff training for crisis situations, noting that the Penetrating Abdominal Trauma Index (P.A.T.I) effectively predicts mortality, advocating for experienced clinical judgement in such emergencies.^52^

In Nigeria, violence related to communal conflicts and other disputes has led to numerous abdominal injuries. The emphasis on early surgical intervention and the hurdles imposed by infrastructural limitations highlight the diversity in complication and mortality outcomes.^4^ Additionally, research in this field within Syria pointed out the shortcomings of initial surgical procedures on violence victims, necessitating further surgeries upon patient transfer to Israel, stressing the importance of effective, yet cost‐efficient surgical approaches in conflict zones.^6^ Despite the potential of nonsurgical interventions in managing abdominal trauma, the need for a detailed understanding of these alternatives remains, as initial inadequate interventions have been linked to fatal outcomes.^6^ This highlights the critical gaps in knowledge regarding nonsurgical treatment options.

## 5 POSITIVE ADVANCEMENTS AND OUTCOMES IN THE MANAGEMENT OF ABDOMINAL TRAUMA WITHIN CONFLICT AND WAR ZONES

The management of abdominal trauma in conflict zones has made significant strides in improving survival rates and functional recovery, highlighting the critical role of surgical interventions and advancements in diagnostics. Procedures such as exploratory laparotomy, damage control surgery (DCS), and specific techniques like hemicolectomy and temporary loop colostomy are pivotal, particularly in cases of penetrating trauma, demonstrating improved outcomes and reduced postoperative mortality.^6^, ^30^, ^36^, ^53^

DCS, focusing on haemorrhage control and contamination management, and damage control laparotomy (DCL), have revolutionised treatment in severe cases, leading to lower mortality rates and less frequent need for faecal diversion.^28^, ^54^ These approaches are particularly effective in military hospitals, evidencing the adaptability of surgical techniques to the demands of combat trauma care. The treatment of colon, liver, and spleen injuries illustrates the evolving landscape of trauma care, moving towards less invasive and more strategic interventions. Historical practices and recent advancements support primary repair of colon wounds under certain conditions and minimally invasive techniques for liver and spleen injuries, reflecting a shift towards procedures that minimise patient trauma and expedite recovery.^55^, ^56^

Diagnostic advancements, including the focused assessment with sonography for trauma (FAST), CT scans, and other imaging techniques, are crucial for rapid injury identification and treatment, significantly enhancing patient outcomes and reducing hospital stay.^4^, ^5^, ^37^, ^52^, ^57^ These tools, alongside adjunctive treatments like airway management and haemorrhage control, play an integral role in streamlining patient recovery and optimising resource use in conflict settings.^47^ Additionally, studies have underscored the importance of timely surgical response and comprehensive postoperative care, especially for patients with high injury severity scores.^43^ Experience from past conflicts informs current practices in rectal trauma management, advocating for proximal diverting colostomy and meticulous cleaning of the injury site.^58^ The necessity of relaparotomy for complications underscores the dynamic and responsive nature of trauma care in such challenging environments.^59^

Furthermore, the success of nonsurgical management in select cases of liver and spleen injuries through external drainage highlights the potential of conservative treatment strategies, advocating for further research and documentation.^4^, ^37^ This holistic approach, blending surgical, diagnostic, and nonsurgical methods, exemplifies the comprehensive nature of trauma care in war‐affected areas, aimed at augmenting patient outcomes through a synergy of rapid intervention, advanced diagnostics, and carefully chosen management strategies.

## 6 STRATEGIES FOR IMPROVING ABDOMINAL TRAUMA CARE IN CONFLICT AND WAR‐AFFECTED LMICS

### 6.1 Expansion of trauma specialist workforce and others

It is projected that LMICs will face a deficit of 10 million health workers by 2030, a shortage that is likely to be exacerbated by conflict.^60^, ^61^ Adequate access to medical professionals is critical for improving outcomes for abdominal trauma victims in conflict‐affected regions.^6^ Policymakers must ensure that resources and funds are adequately allocated to health personnel maintenance and procedural equipment acquisition rather than being diverted for military expenditures.^15^

The development of distance courses, mentorship programmes, and workshops can facilitate the acquisition and maintenance of surgical skills like exploratory laparotomy and damage control surgery and nonsurgical trauma treatment competencies such as external drainage, debridement, and basic first aid skills. This will enable the training of new trauma specialists and improve the skill set of existing personnel. Evidence from the Ukrainian conflict indicates that comprehensive application of damage control techniques improves survival rates at all levels of medical care.^62^

Increasing the number of skilled healthcare personnel would improve the effective management of trauma patients in conflict zones, thereby improving survival rates.^6^, ^30^, ^53^ An expanded, skilled workforce would facilitate prompt surgical treatments, promote faster recovery, and shorten hospital stays, thus enabling patients to resume normal activities more rapidly.^52^, ^53^ Healthcare providers in conflict zones face significant risk, and the provision of appropriate security measures is critical.^14^ Ensuring the safety of healthcare workers and providing appropriate financial compensation may mitigate healthcare worker attrition due to migration from conflict zones.^15^, ^16^ These measures can thus ensure that healthcare staff are equipped and empowered to safely deliver life‐saving care to victims of war, thereby increasing survival rates and mitigating the toll of conflict.

The management of high‐energy war trauma presents unique challenges distinct from those encountered in low‐energy civilian trauma settings. War trauma typically involves injuries from improvised explosive devices, high‐velocity gunshots, and blasts, leading to complex trauma patterns such as extensive soft tissue damage, significant bony destruction, and widespread contamination with environmental debris.^63^ These conditions significantly increase the risk of infections and necessitate rigorous infection control measures.^64^ Moreover, the polytrauma nature of these injuries often involves multiple organ systems, requiring a wide array of surgical interventions and complex, multi‐stage reconstructive procedures.^64^

In contrast, civilian trauma in urban settings generally results in less severe, more localised injuries with lower contamination levels.^65^, ^66^ Consequently, civilian trauma centres do not experience the high volume and variety of severe cases seen in military settings. This discrepancy poses significant challenges in the training of trauma surgeons. Civilian trauma surgeons may not be exposed to the full spectrum of high‐energy injuries, potentially limiting their preparedness for war‐zone deployments.^63^, ^65^, ^66^, ^67^

Maintaining surgical proficiency in high‐energy trauma management is a concern for surgeons transitioning from civilian practice to war zones.^66^ Battlefield injuries often bring out the best in attending surgeons.^63^ However, civilian trauma centres may not provide the same exposure to high‐energy trauma cases, hindering skill development for managing such injuries. For example, studies have shown that the volume of penetrating abdominal injuries treated during military deployments can equate to 3 years of trauma surgery experience in civilian settings.^68^, ^69^

Training for war‐bound trauma surgeons should ideally occur in war‐zone trauma centres to ensure they gain direct experience with the types of injuries they will encounter.^66^, ^67^, ^69^ Civilian trauma centres may not offer sufficient exposure to procedures common in war settings, such as thoracotomy, craniotomy, nephrectomy, and IVC repair.^63^ Furthermore, military medical installations and military surgeons play a crucial role in conflict zones, especially as many civilian doctors evacuate these areas for safety. Military medical facilities must be equipped to handle the unique clinical challenges of high‐energy war trauma, and military surgeons must be trained to operate in resource‐limited, high‐stress environments.^63^

### 6.2 Infrastructural improvements for trauma and emergency surgical care

The availability of robust healthcare infrastructure plays an important role in efficient trauma care and emergency surgical management. Considerable investment is required to improve facilities such as trauma and emergency surgical centres, along with diagnostic centres.

Firstly, financial investment for infrastructural improvements, such as the development of fortified underground emergency and trauma clinics equipped with telemedicine technology, is vital. These installations should withstand the violence of war and conflict, such as shootings and missile attacks. For instance, an Israeli hospital transitioned a 1000‐bed facility into a fortified underground emergency hospital (FUEH), securing its operational readiness for emergencies ranging from warfare to pandemics.^70^

The safe transportation and distribution of medical equipment and resources contributed by assistance organisations and charities, with the implementation of security measures, is essential, as emphasised in Safeguarding Health in Conflict (2016).^14^ This support enables surgical professionals and other trauma multi‐disciplinary teams to perform serial clinical and radiological examinations on patients suspected of abdominal trauma, facilitating precise and effective treatment while ensuring accurate diagnosis and seamless treatment through the secure transportation of vital resources. Timely access to medical and surgical intervention aids rapid emergency response, reducing morbidity and mortality.^6^, ^25^, ^64^ Policymakers should allocate resources to the development of safe, efficient, and resilient transportation networks for medical facilities. Ensuring unimpeded access to these routes can prevent civilian trauma patients from being barred from accessing healthcare institutions.^14^ This measure mitigates the safety risks associated with travel in conflict zones and ensures access to healthcare facilities for timely intervention, potentially leading to improved clinical outcomes.

Additionally, the provision of basic essentials such as water and electricity is critical within conflict zones. Despite recurrent disruptions, preserving these essential amenities is key for healthcare workers to effectively use their resources and infrastructure, thereby enabling effective delivery of life‐saving care for victims of abdominal trauma.^19^, ^20^, ^21^, ^22^, ^23^, ^24^

### 6.3 Encouraging increased humanitarian aid from the international community

The workforce and healthcare infrastructure shortages in LMICs are further exacerbated by the destructive nature of war and conflict. Consequently, it is critical to advocate for concerted efforts from Nongovernmental organisations (NGOs) such as Médecins Sans Frontières, International Medical Corps, and the United Nations to increase resource mobilisation and foster robust funding networks through comprehensive international collaboration.

Primarily, a surge in healthcare volunteers and the provision of medical aid can increase workforce numbers, thereby facilitating prompt access to abdominal trauma treatment and consequently improving clinical outcomes for patients.^6^

Secondly, robust collaborative networks could catalyse the strategies delineated above. Such networks support the acquisition of current diagnostic tools and operational equipment, thus ensuring precise and reliable diagnoses and favourable patient outcomes.^18^, ^26^, ^27^ Moreover, collaborative efforts encourage the exchange of knowledge and skills that can strengthen existing mentorship programmes designed to improve the expertise of local trauma specialists. For instance, an interactive teleconference course offered by a US University successfully certified 12 Iraqi physicians in paediatric advanced life support.^22^

In addition, NGOs could dedicate resources towards the implementation of security measures, ensuring healthcare professionals can safely deliver care and that patients, along with medical aid such as blood products and specialised wound care materials, are transported securely to civilian critical care facilities. Increased physician availability, medical supply sufficiency, and prompt diagnosis collectively contribute to mortality rate reduction.^31^, ^33^ The recurrent violation of international laws advocating for the protection of healthcare workers and facilities during conflict calls for more stringent regulations and consequential penalties.^34^, ^35^, ^71^, ^72^

Additionally, NGOs could contribute their experience and resources towards the establishment of long‐term follow‐up programmes for abdominal trauma patients, facilitating the monitoring of their physical and mental well‐being. Mental health disorders, such as posttraumatic stress disorder (PTSD), which are frequently overlooked, can have lifelong impacts on war victims.^73^ Programmes using evidence‐based treatments like cognitive behavioural therapy (CBT) could mitigate long‐term trauma in these individuals. Emerging strategies such as internet‐delivered CBT for PTSD can address financial and safety concerns related to travel for both international and local mental health practitioners.^74^

### 6.4 Bridging the emergency and trauma care research gaps

Collaborative research efforts between academic clinicians and scientists are vital for the development of cost‐effective technology and management methods. It is essential to leverage the first‐hand experiences of clinicians providing care in conflict‐affected nations, incorporating their understanding of the principal challenges and specific needs of war‐related abdominal trauma cases.^12^ Their insights can guide the development of affordable technologies and strategies to lessen the impact of conflict on abdominal injuries, improving clinical outcomes. Moreover, the participation of local clinicians in data collection can facilitate research progress, which is often impeded due to inadequate data.^6^, ^15^, ^35^ A standardised data collection approach would promote study comparability, thereby catalysing research advancements.^36^, ^38^

Within conflict‐affected settings, systematic audits are essential to identify areas for improvement and guide the development of standardised, cost‐effective, time‐efficient, and evidence‐based protocols and guidelines. Standardisation minimises heterogeneity across healthcare systems and facilitates effective care delivery due to the absence of institutional disparity, fostering cooperation between local and international healthcare professionals providing abdominal trauma care.^36^, ^37^, ^38^

Successful implementation of optimal practices and guidelines necessitates a cooperative local approach involving a multidisciplinary team (MDT) encompassing various specialties like gastrointestinal surgery, intensive care, emergency medicine, nursing, and rehabilitation. This encourages the establishment of a robust healthcare system with swift emergency response and effective trauma management protocols, reducing the overall incidence of abdominal trauma.^25^

Given the inherently destructive nature of war and conflict zones, research into affordable and resistant infrastructural solutions is critical. This includes preparedness for events like bombings, shootings, and potential incursions by hostile entities, ensuring continuity of adequate care for trauma patients in conflict settings.^71^ Finally, telemedicine's advancement within the context of conflicts warrants further research. Considering the potential for disruptions to telecommunication tools and internet services, the creation of durable telecommunication solutions is critical. Such systems should be user‐friendly, enabling widespread civilian adoption and ensuring telemedicine services’ availability as needed.^75^

These aforementioned strategies for improving abdominal trauma care in Conflict‐Affected LMICs have been outlined in Table 3.

**Table 3:** Strategies for Improving Abdominal Trauma Care in Conflict‐Affected Low and Middle Income Countries.

Strategy category	Key initiatives	Description
Expansion of workforce^6^, ^15^, ^30^, ^52^, ^53^, ^60^, ^61^, ^62^, ^63^, ^64^, ^65^, ^66^, ^67^, ^68^, ^69^	Resource allocation to health personnel. Development of training programs. Security and financial incentives for healthcare workers. Train war‐bound trauma surgeons in war‐zone trauma centres.	Addressing the healthcare worker shortage by allocating resources more effectively, enhancing training through distance courses and mentorship, and improving worker safety and compensation.
Infrastructural improvements^6^, ^14^, ^19^, ^20^, ^21^, ^22^, ^23^, ^24^, ^25^, ^70^	Investment in healthcare facilities. Development of fortified clinics. Improving safe transportation and distribution of medical resources. Ensuring the availability of basic essentials like water and electricity.	Upgrading healthcare infrastructure with fortified clinics equipped with telemedicine, ensuring the safe transport of supplies, and maintaining essential services to enable effective trauma care.
Increased humanitarian aid^6^, ^18^, ^22^, ^26^, ^27^, ^31^, ^33^, ^34^, ^35^, ^72^, ^73^, ^74^	Advocacy for more NGO involvement. Surge in healthcare volunteers and medical aid. Collaborative networks for resource mobilisation. Mental health support programs.	Encouraging the international community and NGOs to provide more aid and resources, thereby increasing workforce numbers and improving infrastructure, alongside addressing mental health needs with programs like CBT.
Bridging research gaps^6^, ^12^, ^15^, ^35^, ^36^, ^37^, ^38^, ^71^, ^75^	Collaborative research efforts. Standardised data collection. Development of cost‐effective technologies. Research into resilient infrastructural solutions and telemedicine.	Fostering collaborative research to develop affordable and effective management strategies, standardising data collection, and exploring durable infrastructural and telecommunication solutions for conflict settings.

Abbreviations: CBT, Cognitive Behavioural Therapy; NGO, Nongovernmental Organization.

## 7 LIMITATIONS

There are several limitations in this review that need to be acknowledged. First, the literature search was restricted to certain databases, namely PubMed, EMBASE, Google Scholar, the Cochrane Library, and Scopus. This narrow focus could result in missing relevant research from other databases. Additionally, by excluding publications not in English, important findings published in other languages might be overlooked, which could lead to a loss of crucial data and limit the review's breadth and generalisability. Another significant limitation is the minimal inclusion of studies from regions experiencing war and conflict. This lack of representation might hinder the review's ability to fully address the topic and affect its relevance to various global settings.

## 8 CONCLUSION

Abdominal trauma is a pressing healthcare challenge in conflict‐affected LMICs. The inherent healthcare limitations in LMICs, namely inadequate healthcare workforces, a scarcity of specialised resources, and existing research gaps, are exacerbated under conditions of violence. The volatility of warfare directly threatens physicians, patients, healthcare infrastructures, and basic amenities such as water and electricity, thereby disrupting the provision of high‐quality abdominal care. Overcoming these challenges requires targeted funding allocations to improve healthcare infrastructure and acquire necessary procedural equipment. Moreover, fostering collaborative efforts is essential for the development of programmes dedicated to training abdominal trauma specialists, establishing robust funding networks, and bridging research gaps. The collective execution of these strategies is important to address the challenges presented and improve outcomes for the management of abdominal trauma in war‐ and conflict‐affected LMICs.

## AUTHOR CONTRIBUTIONS

**Wireko Andrew Awuah**: Conceptualisation; methodology; validation; writing—review and editing; writing—original draft; data curation; supervision. **Joecelyn Kirani Tan**: Conceptualisation; Writing—original draft; Methodology; Writing—review and editing; Validation; Data curation. **Muhammad Hamza Shah**: Methodology; Validation; Writing—original draft; Writing—review and editing; Data curation. **Arjun Ahluwalia**: Methodology; Validation; Writing—review and editing; Writing—original draft; Data curation. **Sakshi Roy**: Data curation; Methodology; Validation; Writing—review and editing; Writing—original draft. **Syed Hasham Ali**: Data curation; Methodology; Validation; Writing—review and editing; Writing—original draft. **Tomas Ferreira**: Data curation; Methodology; Validation; Writing—review and editing; Writing—original draft. **Hareesha Rishab Bharadwaj**: Data curation; Methodology; Validation; Writing—review and editing; Writing—original draft. **Favour Tope Adebusoye**: Data curation; Methodology; Validation; Writing—review and editing; Writing—original draft. **Nicholas Aderinto**: Data curation; Methodology; Validation; Writing—original draft; Writing—review and editing. **Adele Mazzoleni**: Data curation; Methodology; Validation; Writing—review and editing; Writing—original draft. **Toufik Abdul‐Rahman**: Data curation; Methodology; Validation; Writing—review and editing; Writing—original draft. **Denys Ovechkin**: Data curation; Methodology; Validation; Writing—review and editing; Writing—original draft.

## CONFLICT OF INTEREST STATEMENT

The authors declare no conflicts of interest.

## TRANSPARENCY STATEMENT

The lead author Wireko Andrew Awuah affirms that this manuscript is an honest, accurate, and transparent account of the study being reported; that no important aspects of the study have been omitted; and that any discrepancies from the study as planned (and, if relevant, registered) have been explained.

## DECLARATIONS

All authors have read and approved the final version of the manuscript. WAA had full access to all of the data in this study and takes complete responsibility for the integrity of the data and the accuracy of the data analysis.

## Data Availability

Data sharing is not applicable to this article as no new data were created or analyzed in this study.

## References

[1] Krug EG , Mercy JA , Dahlberg LL , Zwi AB . The world report on violence and health. Lancet. 2002;360:1083–1088. 12384003 10.1016/S0140-6736(02)11133-0

[2] Jawad M , Hone T , Vamos EP , Roderick P , Sullivan R , Millett C . Estimating indirect mortality impacts of armed conflict in civilian populations: panel regression analyses of 193 countries, 1990‐2017. BMC Med. 2020;18:266. 32907570 10.1186/s12916-020-01708-5 PMC7487992

[3] Martin GJ , Dunne JR , Cho JM , Solomkin JS . Prevention of infections associated with combat‐related thoracic and abdominal cavity injuries. J Trauma Injury Infection Critic Care. 2011;71:S270–S281. 10.1097/TA.0b013e318227adae 21814093

[4] Ojo E , Ozoilo K , Sule A , et al. Abdominal injuries in communal crises: the jos experience. J Emerg Trauma Shock. 2016;9:3–9. 26957819 10.4103/0974-2700.173867 PMC4766761

[5] Alsaid B , Alhimyar M , Alnweilaty A , et al. Laparotomy due to War‐Related penetrating abdominal trauma in civilians: experience from Syria 2011‐2017. Disaster Med Public Health Prep. 2021;15:615–623. 32489173 10.1017/dmp.2020.77

[6] Bickel A , Akinichev K , Weiss M , et al. Challenges in abdominal re‐exploration for war casualties following on‐site abdominal trauma surgery and subsequent delayed arrival to definitive medical care abroad ‐ an unusual scenario. BMC Emerg Med. 2022;22:132. 35850737 10.1186/s12873-022-00687-5 PMC9295351

[7] Rignault DP . Abdominal trauma in war. World J Surg. 1992;16:940–946. 1462634 10.1007/BF02066996

[8] Hildebrand F , Pape HC , Horst K , et al. Impact of age on the clinical outcomes of major trauma. Eur J Trauma Emerg Surg. 2016;42:317–332. 26253883 10.1007/s00068-015-0557-1

[9] Ologunde R , Le G , Turner J , et al. Do trauma courses change practice? A qualitative review of 20 courses in East, Central and Southern Africa. Injury. 2017;48:2010–2016. 28625562 10.1016/j.injury.2017.06.007

[10] Gauss T , Merckx P , Brasher C , et al. Deviation from a preoperative surgical and anaesthetic care plan is associated with increased risk of adverse intraoperative events in major abdominal surgery. Langenbecks Arch Surg. 2013;398:277–285. 23149461 10.1007/s00423-012-1028-3

[11] Bouzat P , Valdenaire G , Gauss T , et al. Early management of severe abdominal trauma. Anaesthesia Critic Care Pain Med. 2020;39:269–277. 10.1016/j.accpm.2019.12.001 31843714

[12] Blyth DM , Yun HC , Tribble DR , Murray CK . Lessons of war: combat‐related injury infections during the Vietnam war and operation Iraqi and enduring freedom. J Trauma Acute Care Surg. 2015;79:S227–S235. 26406435 10.1097/TA.0000000000000768 PMC4586048

[13] Woodward A , Sheahan K , Martineau T , Sondorp E . Health systems research in fragile and conflict affected states: a qualitative study of associated challenges. Health Res Policy Syst. 2017;15:44. 28592283 10.1186/s12961-017-0204-x PMC5461673

[14] Safeguarding Health in Conflict Coalition . December 20, 2023. https://www.safeguardinghealth.org/members

[15] Ali Jadoo SA , Aljunid SM , Dastan I , et al. Job satisfaction and turnover intention among Iraqi doctors‐‐a descriptive cross‐sectional multicentre study. Hum Resour Health. 2015;13:21. 25903757 10.1186/s12960-015-0014-6 PMC4407309

[16] Bou‐Karroum L , Daou KN , Nomier M , et al. Health care workers in the setting of the “Arab Spring”: a scoping review for the Lancet‐AUB commission on Syria. J Glob Health. 2019;9:010402. 30410745 10.7189/jogh.09.010402 PMC6207103

[17] Marks IH , Kanya L , Singh D , Saleh R , Friebel R , Hargest R . O107 surgical workforce in conflict: qualitative perspectives from the Middle East and North Africa. Br J Surg. 2023;110(suppl ment_3):znad101.107. 10.1093/bjs/znad101.107 41369652

[18] Ogbuanya AUO , Ugwu NB , Kwento N , Enyanwuma EI , Anyigor FF , Oko U . Abdominal injuries from civilian conflicts: an emerging global health challenge in rural southeast Nigeria. Ann Glob Health. 2023;89:4. 36743283 10.5334/aogh.3973 PMC9881438

[19] Sheibani BIMA , Hadi NR , Hasoon T . Iraq lacks facilities and expertise in emergency Medicine. BMJ. 2006;333:847. 17053243 10.1136/bmj.38986.476782.68 PMC1618447

[20] Fouad FM , Sparrow A , Tarakji A , et al. Health workers and the weaponisation of health care in Syria: a preliminary inquiry for the Lancet‐American University of Beirut commission on Syria. Lancet. 2017;390:2516–2526. 28314568 10.1016/S0140-6736(17)30741-9

[21] Afzal MH , Jafar AJN . A scoping review of the wider and long‐term impacts of attacks on healthcare in conflict zones. Med Confl Surviv. 2019;35:43–64. 30943776 10.1080/13623699.2019.1589687

[22] Donaldson RI , Mulligan DA , Nugent K , et al. Using tele‐education to train civilian physicians in an area of active conflict: certifying Iraqi physicians in pediatric advanced life support from the United States. J Pediatr. 2011;159:507–509.e1. 21722915 10.1016/j.jpeds.2011.05.003

[23] Quinn MM , Smith PM . Gender, work, and health. Ann Work Expo Health. 2018;62:389–392. 29617721 10.1093/annweh/wxy019

[24] Ramalingam T . Extremity injuries remain a high surgical workload in a conflict zone: experiences of a British field hospital in Iraq, 2003. J R Army Med Corps. 2004;150:187–190. 15624410 10.1136/jramc-150-03-06

[25] Lotfollahzadeh S , Burns Bracken , Waheed A . Penetrating Abdominal Trauma. StatPearls; 2019. 29083811

[26] Ayoade BA , Salami BA , Tade AO , et al Abdominal injuries in olabisi onabanjo university teaching hospital sagamu, Nigeria: pattern and outcome. Niger J Othop Trauma. 2006;5:45–49.

[27] Onuminya JE , Ohwowhiagbese E . Pattern of civilian gunshot injuries in irrua, Nigeria. South African J Surg. 2005;43:170–172. 16440592

[28] Smith IM , Naumann DN , Marsden MER , Ballard M , Bowley DM . Scanning and war: utility of FAST and CT in the assessment of battlefield abdominal trauma. Ann Surg. 2015;262:389–396. 25405557 10.1097/SLA.0000000000001002

[29] Habib RR , Halwani DA , Mikati D , Hneiny L . Sex and gender in research on healthcare workers in conflict settings: A scoping review. Int J Environ Res Public Health. 2020;17:4331. 32560496 10.3390/ijerph17124331 PMC7346087

[30] Cardi M , Ibrahim K , Alizai SW , et al. Injury patterns and causes of death in 953 patients with penetrating abdominal war wounds in a civilian independent non‐governmental organization hospital in lashkargah, Afghanistan. World J Emerg Surg. 2019;14:51. 31832085 10.1186/s13017-019-0272-z PMC6868865

[31] van Bergen L . Doctors at war: life and death in a field hospital. Med Confl Surviv. 2017;33:325–327. 29108426 10.1080/13623699.2017.1398962

[32] Leppäniemi AK . Abdominal war wounds‐‐experiences from red cross field hospitals. World J Surg. 2005;29:S67–S71. 15815827 10.1007/s00268-004-2065-z

[33] Opvang DM . Penetrating abdominal war injuries among the war victims at lacor hospital in gulu, Northern Uganda. East Central Afri J Surg. 2001;6(2). https://www.ecajs.org/article/119923-penetrating-abdominal-war-injuries-among-the-war-victims-at-lacor-hospital-in-gulu-northern-uganda

[34] McNeilly B , Jasani G , Cavaliere G , Alfalasi R , Lawner B . The rising threat of terrorist attacks against hospitals. Prehosp Disaster Med. 2022;37:223–229. 35322774 10.1017/S1049023X22000413

[35] Al‐Ashwal FY , Kubas M , Zawiah M , et al. Healthcare workers’ knowledge, preparedness, counselling practices, and perceived barriers to confront COVID‐19: a cross‐sectional study from a war‐torn country, Yemen. PLoS ONE. 2020;15:0243962. 10.1371/journal.pone.0243962 PMC7732096 33306750

[36] Ogwang DM . Penetrating abdominal war injuries among the war victims at lacor hospital in gulu, Northern Uganda. East Central Afri J Surg. 2001;6:71–74.

[37] Sharma A , Agrawal V , Lal A , Choudhury A , Chatterjee P , Ganguly MV . Penetrating abdominal injuries due to firearms in combat zone – single center experience. J Mar Med Soc. 2019;21:139.

[38] Wani I , Parray FQ , Sheikh T , et al. Spectrum of abdominal organ injury in a primary blast type. World J Emerg Surg. 2009;4:46. 20025766 10.1186/1749-7922-4-46 PMC2803452

[39] Bilén K , Ponzer S , Castrén M , Pettersson H , Ottosson C . The impact of trauma mechanism on outcome: a follow‐up study on health‐related quality of life after major trauma. Eur J Trauma Emerg Surg. 2010;36:449–455. 26816226 10.1007/s00068-010-0003-3

[40] Bath MF , Kohler K , Hobbs L , et al. The impact of trauma system implementation on patient quality of life and economic burden: a systematic review study protocol. Int J Surg Protoc. 2023;27:84–89. 36875324 10.29337/ijsp.187 PMC9983497

[41] Hamdawi MA , Nile AK , Abed WM . Penetrating abdominal trauma: evaluation in Baghdad during the pandemic Covid‐19. J Emerg Med Trauma Acute Care. 2022;3:11.

[42] Khuda Bux GM , Harhra NAA , Al Khader IM . Management of abdominal trauma at Al‐Gamhouria general modern Hospital‐Aden‐Yemen. Univ Aden J Nat Appl Sci. 2022;26:241–249.

[43] Agbroko S , Osinowo A , Jeje E , Atoyebi O . Determinants of outcome of abdominal trauma in an urban tertiary center. Niger J Surg. 2019;25:167–171. 31579371 10.4103/njs.NJS_2_19 PMC6771180

[44] Lone RA , Akbar BM , Sharma M , et al. Missile diaphragmatic injuries: kashmir experience. Int J Health Sci. 2009;3:19–21. PMC3068792 21475506

[45] Gumeniuk K , Lurin IA , Tsema I , Malynovska L , Gorobeiko M , Dinets A . Gunshot injury to the colon by expanding bullets in combat patients wounded in hybrid period of the Russian‐Ukrainian war during 2014‐2020. BMC Surg. 2023;23:23. 36707838 10.1186/s12893-023-01919-6 PMC9883919

[46] Zangana AM . Penetrating liver war injury: a report on 676 cases, after Baghdad invasion and Iraqi civilian war April 2003. Adv Med Dent Sci. 2007;10. link.gale.com/apps/doc/A215515446/HRCA?u=anon~54f761ee&sid=googleScholar&xid=12397bd7

[47] Angelici AM , Montesano G , Nasti AG , Palumbo P , Vietri F . Treatment of gunshot wounds to the colon: experience in a rural hospital during the Civil War in Somalia. Ann Ital Chir. 2004;75:461–464. 15754697

[48] Zangana A , Zangana M , Allan A , et al Blunt abdominal trauma after Baghdad invasion April 2003 a report on 1020 patients. Adv Med Dent Sci. 2008;2:28–33.

[49] Iflazoglu N , Ureyen O , Oner OZ , Tusat M , Akcal MA . Complications and risk factors for mortality in penetrating abdominal firearm injuries: analysis of 120 cases. Int J Clin Exp Med. 2015;8:6154–6162. 26131219 PMC4483940

[50] Ugur M , Akkucuk S , Koca YS , Oruc C , Aydogan A . Missed injuries in patients with abdominal gunshot trauma: risk factors and mortality rates. European Surgery. 2016;48:347–351.

[51] Tribble DR , Li P , Warkentien TE , et al. Impact of operational theater on combat and noncombat Trauma‐Related infections. Mil Med. 2016;181:1258–1268. 27753561 10.7205/MILMED-D-15-00368 PMC5123796

[52] Arafat S , Alsabek MB , Ahmad M , Hamo I , Munder E . Penetrating abdominal injuries during the Syrian war: patterns and factors affecting mortality rates. Injury. 2017;48:1054–1057. 28238300 10.1016/j.injury.2017.02.005

[53] Al‐Bahlooli SH , Al‐Amry AL , Al‐Shujaa MA . Civilian gunshot injuries of the abdomen at hajjah governorate in Yemen. Ann Med Health. 2020;2:1–6.

[54] Beekley AC . Damage control resuscitation: a sensible approach to the exsanguinating surgical patient. Crit Care Med. 2008;36:S267–S274. 18594252 10.1097/CCM.0b013e31817da7dc

[55] Morris DS , Sugrue WJ . Abdominal injuries in the war wounded of Afghanistan: a report from the international committee of the red cross hospital in Kabul. J Br Surg 1991;78:1301–1304. 10.1002/bjs.1800781108 1760686

[56] Feliciano DV , Mattox KL , Burch JM , Bitondo CG , Jordan Jr. GL . Packing for control of hepatic hemorrhage. J Trauma Injury Infection Critic Care. 1986;26:738–743. 10.1097/00005373-198608000-00010 3488413

[57] Beck‐Razi N , Fischer D , Michaelson M , Engel A , Gaitini D . The utility of focused assessment with sonography for trauma as a triage tool in multiple‐casualty incidents during the second Lebanon war. J Ultrasound Med. 2007;26:1149–1156. 17715308 10.7863/jum.2007.26.9.1149

[58] Ganchrow MMI . Surgical management of traumatic injuries of the colon and rectum. AArch Surg. 1970;100:515–520. 10.1001/archsurg.1970.01340220191032 5417175

[59] Bosarge PL , Como JJ , Fox N , et al. Management of penetrating extraperitoneal rectal injuries: an eastern association for the surgery of trauma practice management guideline. J Trauma Acute Care Surg. 2016;80:546–551. 26713970 10.1097/TA.0000000000000953

[60] World Health Organization . Health Workforce. January 10, 2024. https://www.who.int/health-topics/health-workforce#tab=tab_1

[61] Saghafinia M , Nafissi N , Motamedi M , et al. Assessment and outcome of 496 penetrating gastrointestinal warfare injuries. J R Army Med Corps. 2010;156:25–27. 20433101 10.1136/jramc-156-01-05

[62] Khomenko I , Shapovalov V , Tsema I , et al. Hydrodynamic rupture of liver in combat patient: a case of successful application of “damage control” tactic in area of the hybrid war in east Ukraine. Surg Case Rep. 2017;3:88. 28812283 10.1186/s40792-017-0363-6 PMC5557719

[63] Shabhay AA , Shabhay ZA , Mwami AS , Massaga FA . Do urban city trauma centers suffice as Pre‐Deployment training and Post‐Deployment skills retaining centers? Disaster Med Public Health Prep. 2023;17:e526. 37946536 10.1017/dmp.2023.193

[64] Bhandari PS , Mukherjee MK , Maurya S . Reconstructive challenges in war wounds. Indian J Plastic Surg. 2012;45:332–339. 10.4103/0970-0358.101316 PMC3495384 23162233

[65] Truitt MS , Johnson V , Rivera M , Mangram A , Lorenzo M , Dunn E . Civilian and military trauma: does civilian training prepare surgeons for the battlefield? Am Surg. 2011;77:19–21. 21396297

[66] Giannou C . A volunteer surgeon in war zones: experience of 35 years and a call to action. Current Trauma Reports. 2017;3:75–77. 28303215 10.1007/s40719-017-0075-1 PMC5332512

[67] Uchino H , Kong VY , Bruce JL , et al. Preparing Japanese surgeons for potential mass casualty situations will require innovative and systematic programs. Eur J Trauma Emergency Surg. 2019;45:139–144. 10.1007/s00068-017-0871-x 29119221

[68] Hoencamp R , Tan ECTH , Idenburg F , et al. Challenges in the training of military surgeons: experiences from Dutch combat operations in Southern Afghanistan. Eur J Trauma Emerg Surg. 2014;40:421–428. 26816237 10.1007/s00068-014-0401-z

[69] Ramasamy A , Hinsley DE , Edwards DS , Stewart MPM , Midwinter M , Parker PJ . Skill sets and competencies for the modern military surgeon: lessons from UK military operations in Southern Afghanistan. Injury. 2010;41:453–459. 20022003 10.1016/j.injury.2009.11.012

[70] Halberthal, MD, MHA M , Berger, MD G , Hussein, MD K , et al. Israeli underground hospital conversion for treating COVID‐19 patients. Am J Disaster Med. 2020;15:159–167. 33270207 10.5055/ajdm.2020.0371

[71] No Protection, No Respect: Health Workers and Health Facilities Under Attack: 2015 and Early 2016. Retrieved: 2024, January 10

[72] Usman S , Shah MH , Roy S , Ahluwalia A . Breaking silence in the shadows of conflict: ethical challenges and policy imperatives for medical professionals reporting human rights breaches in militarized zones. Med Confl Surviv. 2024;40:44–52. 38213005 10.1080/13623699.2023.2301193

[73] Lis‐Turlejska M , Luszczynska A , Plichta A , Benight CC . Jewish and non‐jewish world war II child and adolescent survivors at 60 years after war: effects of parental loss and age at exposure on well‐being. Am J Orthopsychiatry. 2008;78:369–377. 19123756 10.1037/a0014166

[74] Young C , Campbell K Internet‐Delivered Cognitive Behavioral Therapy for Post‐Traumatic Stress Disorder: A Review of Clinical Effectiveness. Canada, Canadian Agency for Drugs and Technologies in Health, 2018. 30896898

[75] Merrell RC , Cone SW , Rafiq A . Telemedicine in extreme conditions: disasters, war, remote sites. Stud Health Technol Inform. 2008;131:99–116. 18305326

